# *Wolbachia* Utilize Host Actin for Efficient Maternal Transmission in *Drosophila melanogaster*

**DOI:** 10.1371/journal.ppat.1004798

**Published:** 2015-04-23

**Authors:** Irene L. G. Newton, Oleksandr Savytskyy, Kathy B. Sheehan

**Affiliations:** Department of Biology, Indiana University, Bloomington, Indiana, United States of America; Stanford University, UNITED STATES

## Abstract

*Wolbachia pipientis* is a ubiquitous, maternally transmitted bacterium that infects the germline of insect hosts. Estimates are that *Wolbachia* infect nearly 40% of insect species on the planet, making it the most prevalent infection on Earth. The bacterium, infamous for the reproductive phenotypes it induces in arthropod hosts, has risen to recent prominence due to its use in vector control. *Wolbachia* infection prevents the colonization of vectors by RNA viruses, including *Drosophila* C virus and important human pathogens such as Dengue and Chikungunya. Here we present data indicating that *Wolbachia* utilize the host actin cytoskeleton during oogenesis for persistence within and transmission between *Drosophila melanogaster* generations. We show that phenotypically wild type flies heterozygous for cytoskeletal mutations in *Drosophila* profilin (*chic^221^*/+ and *chic^1320^*/+) or villin (*qua^6-396^*/+) either clear a *Wolbachia* infection, or result in significantly reduced infection levels. This reduction of *Wolbachia* is supported by PCR evidence, Western blot results and cytological examination. This phenotype is unlikely to be the result of maternal loading defects, defects in oocyte polarization, or germline stem cell proliferation, as the flies are phenotypically wild type in egg size, shape, and number. Importantly, however, heterozygous mutant flies exhibit decreased total G-actin in the ovary, compared to control flies and *chic^221^* heterozygous mutants exhibit decreased expression of profilin. Additionally, RNAi knockdown of profilin during development decreases *Wolbachia* titers. We analyze evidence in support of alternative theories to explain this *Wolbachia* phenotype and conclude that our results support the hypothesis that *Wolbachia* utilize the actin skeleton for efficient transmission and maintenance within *Drosophila*.

## Author Summary

The world’s most common intracellular infection, *Wolbachia pipientis*, infects 40% of insect species and is currently used to prevent transmission of Dengue by mosquitoes. The bacterium targets the germline of insects, where it is faithfully transmitted to the developing oocyte and the next generation. Here we identify host cytoskeletal proteins required by *Wolbachia* in order to be efficiently transmitted between *Drosophila melanogaster* generations. We show that after only two generations in a phenotypically wild type, heterozygous mutant fly, *Wolbachia* infections are cleared or reduced in titer. Characterization of the mutants suggests that *Wolbachia* is sensitive to the regulation of actin in the ovary and that actin may be used by *Wolbachia* to both target and proliferate within host tissues and to be faithfully, maternally transmitted.

## Introduction

*Wolbachia pipientis* is an intracellular α-proteobacterium that forms symbioses with an extremely broad array of hosts, including isopods, nematodes, and insects [1]. *Wolbachia* were first noted in the tissues of the mosquito, *Culex pipiens*, by Hertig and Wolbach in 1924, but subsequently, many more insects were found to harbor *Wolbachia*. Current estimates suggest that upwards of 40% of insect species may be infected by the parasite, making *Wolbachia* one of the most common intracellular bacteria on the planet [2]. *Wolbachia* are well known for the reproductive effects induced in the host, which range from the exotic (male killing) to the most common of reproductive effects, cytoplasmic incompatibility (CI) [1]. This recalcitrant, obligate symbiont has received much attention recently due to medical relevance. *Wolbachia* are heavily studied as potential drug targets for filarial nematode infection [3,4] and are currently being implemented to prevent transmission of Dengue fever from mosquitoes to humans [5,6]. *Wolbachia* may be one answer to controlling some vector borne human diseases—indeed mosquitoes harboring a virus-blocking strain of *Wolbachia* are presently being released in underdeveloped parts of the world with this hope in mind [6–8]. Given the ubiquity of *Wolbachia* in the insect world, and its relevance to human health, it is essential to understand the biological basis of transmission of the symbiont between host generations.

*Wolbachia* are maternally transmitted bacteria that infect the germline of their hosts such that their transmission fidelity in wild populations is extraordinarily high. Although physiologically stressful conditions are known to induce the loss of superinfections [9], perfect transmission has been measured in control laboratory *Drosophila* populations as well as in insects harboring transferred *Wolbachia* infections [10–12]. Localization in the germline, and in the developing oocyte, is critical to *Wolbachia’s* maternal transmission and in addition, densities in the embryo, and posterior localization, are correlated with reproductive phenotype (e.g. CI) [13,14].

Previous studies have provided some support for *Wolbachia* interactions with host cytoskeletal elements. Specifically, in *Drosophila*, *Wolbachia* require host microtubules and the motors Dynein and Dynactin for anterior localization early in development and Kinesin-1 for posterior localization in mid oogenesis, positioning them for inclusion in the germline [15,16]. This localization is thought to be crucial to the bacterium’s faithful transmission to subsequent generations at the appropriate densities. Additionally, *Wolbachia* use astral microtubules during asymmetric divisions in the developing embryo, leading to the widespread, but uneven, pattern of localization of the bacteria in adult tissues [17]. In both worms and flies, *Wolbachia* undergo somatic cell to germline transmission, suggesting an ability for the bacterium to alter the host actin cytoskeleton to facilitate uptake by germ cells [18,19]. More recently, work has suggested interactions between *Wolbachia* proteins from the *Brugia malayi* symbiont and host actin [20], although *Wolbachia* ultrastructure in *Brugia* does not reveal any obvious mechanism (such as actin comet tails produced during infection in other *Rickettsiales*) [21]. These previous studies have relied on microscopy and *in vitro* biochemistry and until now, no genetic evidence of interaction between *Wolbachia* and actin has been reported.

Here we present data showing that *Wolbachia* persistence and transmission within *Drosophila melanogaster* is sensitive to mutations affecting the actin cytoskeleton. The importance of actin during *Wolbachia* infection was investigated by acquiring *Drosophila* mutants in actin binding proteins, both involved in the regulation of F-actin filaments: the homologs of profilin (chickadee), which regulates the formation of filamentous actin, and villin (quail), which bundles actin filaments. We show that flies heterozygous for mutations in profilin (*chic* ^*221*^/+ and *chic* ^*1320*^/+) or villin (*qua* ^*6-396*^/+) lose *Wolbachia* infection after only a few generations. Importantly, the effect is due to *both* an inability of *Wolbachia* to efficiently colonize germaria in heterozygous mutant hosts and by a reduction in titer when the host is infected. Importantly, both the less severe *chic* allele (*chic* ^*1320*^), known to decrease an oocyte specific isoform of *Drosophila* profilin *chickadee* [22], as well as the null *chic* allele (*chic* ^*221*^) produced a *Wolbachia* titer phenotype. We identified two different actin binding proteins (profilin and villin) that affect *Wolbachia* transmission and maintenance, supporting the conclusion that *Wolbachia* persistence within the host is sensitive to actin.

## Materials and Methods

### *Drosophila* stocks

Standard methods were used for all crosses and culturing. The following stocks were obtained from the Bloomington Drosophila Stock Center (BDSC) at Indiana University (http://flystocks.bio.indiana.edu/): stock number 145, which carries *w* ^*1*^ was used as the *Wolbachia* infected control line. Two *chickadee* mutant fly stocks were used in this study. The *chic* ^*221*^ *cn* ^*1*^ */CyO; ry* ^*506*^ flies carry a null recessive allele resulting from the deletion of 5’ non-coding and some *chic*-coding sequences [22]. The *P{PZ}chic* ^*01320*^ *cn* ^*1*^ */CyO; ry* ^*506*^ flies carry a strong homozygous infertile loss-of-function allele in *chickadee*, generated by P-element insertion [23]. The *quail* mutant flies, *qua* ^*6-396*^ */SM1*, carry a female sterile, recessive mutation induced by ethyl methanesulfonate [24]. We also utilized two chromosomal deficiency stocks: #9507, *w* ^*1118*^; Df(2L)BSC148/CyO, is a chromosomal deletion of segments 36C8-36E3, covering the region containing the *quail* locus. The second of these stocks #24377, *w* ^*1118*^; Df(2L)BSC353/CyO, covers segments 26A3-26B3, the region containing the *chic* locus. Both of these chromosomal deletions are part of the aberration stock collection and were created by FLP-mediated recombination between FRT-bearing transposon insertions [25]. *Wolbachia* were introduced into the heterozygous mutant backgrounds through crosses between *w* ^*1*^ infected females (stock 145) and uninfected heterozygous males (mutant/*CyO*). In order to control for genetic background, we also created isogenized lines by backcrossing stock 145 and each mutant line to an uninfected w; Sco/Cyo stock for three generations (as per [26], S1 Fig). We used sibling controls to identify *Wolbachia* titer differences related to genotype.

In addition to these isogenized lines, and to examine the effect on *Wolbachia* titer of profilin knockdown during development, we utilized a fly stock carrying a UAS inducible profilin-specific short hairpin silencing trigger (RNAi; stock #34523, genotype *y* ^*1*^ sc* v ^*1*^; P{TRiP.HMS00550}attP2) [27]. In order to test the effect of induction on fly development (to recapitulate the developmental lethality of the profilin null) we crossed homozygous females from this line to *w;* P{w+, Act GAL4} /TM3 males. In order to knock down profilin, we then crossed homozygous females from this line to a homozygous Hsp70:Gal4 driver (a generous gift from Brian Calvi). An additional control for expression from the Hsp70:Gal4 driver included a UAS:GFP stock (also a gift from Brian Calvi). Flies were shocked at 37C for 10 minutes to induce the short hairpin. *Wobachia* infection status for stocks acquired from the BDSC was determined via PCR and Western blot targeting the gene wsp or its product (see methods below). All flies examined for *Wolbachia* infection in the experiments below were age matched in order to avoid confounding correlations between fly age and *Wolbachia* titer.

### Western blots

Flies were ground in 1.5ml centrifuge tubes using an electric hand drill and disposable pestle in lysis buffer: 150mM NaCl, 1% Triton X-100, 50mM TrisHCl (pH8) containing HALT protease inhibitor cocktail (Thermo Scientific) and 5 mM EDTA. The lysates were centrifuged for 1 minute at 8000 X g to pellet debris. Samples were heated for 5 minutes at 95°C in Laemmli sample buffer containing 5% β-mercaptoethanol (Bio-Rad) prior to SDS-PAGE electrophoresis. Proteins were separated on 4–20% Tris-Glycine NB precast gels (NuSep) in 1X Tris/Glycine/SDS running buffer (Bio-Rad) and transferred to PVDF membrane in Tris-Glycine transfer buffer with 15% methanol at 40v on ice for 3–4 hours. The membrane was blocked for 5 minutes in Starting Block T20 (TBS) Blocking Buffer (Thermo Scientific), followed by incubation in primary antibody (for 1 hour at RT or O/N at 4°C) according to standard protocols. SuperSignal West Pico Chemiluminescent Substrate (Thermo Scientific) was used according to the manufacturer’s instructions to detect HRP (after incubation with secondary antibodies) on the immunoblots. Blots were re-probed after stripping in 100mM Glycine, 0.15 ND-40, 1% SDS, pH 2 for 1 hour at RT, then overnight at 4°C. PageRuler prestained protein ladder (Thermo Scientific) was used as a molecular mass marker. The following antibody was obtained through BEI Resources, NIAID, NIH: Monoclonal Anti-*Wolbachia* Surface Protein (WSP), NR-31029, and used at a dilution of 1:1000. Additionally, we used anti-actin monoclonal at 1:10,000 (Seven Hills Bioreagents) as a loading control as well as secondary antibodies: HRP enzyme conjugates (Invitrogen) at 1:5000. Densitometry measures were made in ImageJ using scanned film with same exposure times across multiple experiments. Control and experimental flies were included on the same blot in order to ensure consistencies in measured ratios.

### Immunohistochemistry, fluorescence in situ hybridization, and microscopy

Immunohistochemistry was performed as follows: ovaries for immunolocalization were dissected in Ringer’s solution 3–5 days after fly eclosion, then fixed as previously described [28] with following modification: 6% formaldehyde devitellinizing buffer was replaced with 5.3% paraformaldehyde in same (Electron Microscopy Sciences). After a series of washes in PBS buffer, ovaries were blocked with 0.5% BSA in PBST for 10 min. The monoclonal anti-Heat Shock Protein 60 (HSP60), clone LK2, H 3524 (Sigma) was diluted 1:150 in PBST with 1% BSA or a custom antibody created against full length *Wolbachia* FtsZ was diluted 1:150 in PBST with 1% BSA. Cy3 conjugated to goat anti-mouse secondary antibody (Jackson Immunoresearch) or rabbit secondary antibody (Jackson Immunoresearch) diluted 1:250 in PBST + BSA was used to detect the primary antibody. For F-actin detection we used Acti-stain 488 Fluorescent Phalloidin (Cytoskeleton, Inc). Tissues were mounted in Slow Fade “Gold” antifade reagent (Invitrogen) and stored at 4°C.

To confirm staining by immunohistochemistry, we also used fluorescent *in situ* hybridization, following published protocols [18] with the following modifications: post-fixation in 4% paraformaldehyde in DEPC treated PBS, ovaries were dehydrated in methanol and stored overnight at -20**°**C. In the morning, washes in DEPC-PBST preceded a 5 minute proteinase K treatment (0.05 mg/mL) at 37C before prehybridization in hyb buffer (50% formamide, 5X SSC, 250 mg/L SS DNA, 0.5x Denhardts, 20 mM Tris-HCl and 0.1% SDS). Universal bacterial probe EUB338 conjugated to Alexa488 (Molecular Probes) was used to detect *Wolbachia* in the ovarioles. Hybridized ovaries were mounted in Slow Fade “Gold” antifade reagent (Invitrogen).

Images were taken as Z-series stacks at 1.5 um intervals using a Nikon E800 fluorescent microscope with 40x oil objective and processed using Metamorph imaging software (Molecular Devices). Care was taken such that exposure times were normalized across all experiments. For quantification of *Wolbachia* and F-actin within the germarium z-sections maximum projections were used and regions of the germarium demarcated using masks (S2 Fig). We were careful to exclude the peritoneal sheath for F-actin quantification and for Z-stacks where the sheath was difficult to exclude (due to placement of the sections), the images were not included in the F-actin quantification. Germaria showing aggregates of *Wolbachia* were scored based on a striking pixel intensity in the presumed somatic stem cell niche.

### DNA and RNA extractions and polymerase chain reactions

DNA was extracted from flies utilizing the Qiagen DNeasy Blood and Tissue Kit (Qiagen) according to directions with the following modification. Flies were ground in a 1.5ml centrifuge tube using a disposable pestle and an electric hand drill in 180 ul PBS, 200 ul ALT buffer, and 20 ul Proteinase K solution. The samples were incubated at 56°C for 10 minutes with vigorous shaking and then centrifuged briefly to pellet debris before continuing with the ethanol precipitation in the kit protocol. DNAs were quantified by measuring absorbance at 260nm using an Epoch spectrophotometer (Biotek). Semi-quantitative PCR was performed by standardizing the amount of DNA in each reaction. We utilized Phusion High Fidelity PCR Master Mix with HF buffer (New England Biolabs). The protocol for amplification was: 98°C for 3 minutes, followed by 25 cycles of 98°C for 10 seconds, 56°C for 45 seconds, 72°C for 1 minute 30 seconds with a final 10 minute extension at 72°C. Primers were as follows: wsp F1 5’-GTC CAA TAR STG ATG ARG AAA C—3’ and wsp R1 5’- CYG CAC CAA YAG YRC TRT AAA -3’ [29]. RNA and DNA were extracted from individual flies or pupae using a modified Trizol extraction protocol. Briefly, 500 uL of Trizol was added to flies and samples homogenized using a pestle. After a 5 minute incubation at room temperature, a 12,000 rcf centrifugation (at 4C for 10 min) was followed by a chloroform extraction. Aqueous phase containing RNA was extracted a second time with phenol:chloroform before isopropanol precipitation of RNA. This RNA pellet was washed and resuspended in The RNA Storage Solution (Ambion). DNA extraction from the same flies or pupae was performed using ethanol precipitation of the organic phase during the first chloroform extraction. Quantitative PCR was performed on the DNA to detect the *Wolbachia* titer (with reference to the host) using an Applied Biosystems StepOne Real-time PCR system and SybrGreen chemistry (Applied Biosystems). We used wsp primers for *Wolbachia* (Forward: CATTGGTGTTGGTGTTGGTG; Reverse: ACCGAAATAACGAGCTCCAG) and Rpl32 primers for the host (Forward: CCGCTTCAAGGGACAGTATC; Reverse: CAATCTCCTTGCGCTTCTTG) at the following temperatures: 95**°**C for 10 min, then 40 cycles of 95**°**C for 15 seconds and 60**°**C for 1 minute. To detect number of profilin transcripts we utilized the RNA extracted from these flies and the SensiFAST SYBER Hi-ROX One-step RT mix (Bioline) and the following primer set: chicF: TGCACTGCATGAAGACAACA, chicR: GTTTCTCTACCACGGAAGCG (FlyPrimerBank, DRSC). Reactions were performed in a 96-well plate and calibration standards were used in every run to calculate primer efficiencies. These efficiencies, along with the CT values generated by the machine, were used to calculate the relative amounts of *Wolbachia* using the ΔΔ Ct (Livak) and Pfaffl methods [30].

### F and G-actin quantification

In order to identify the ratio of filamentous to globular actin in ovaries from age matched flies, we used ultracentrifugation coupled to SDS-PAGE and Western blots using an *in vivo* F/G actin assay kit (Cytoskeleton, Inc). Age-matched, virgin female flies from *chic* ^*221*^ */Cyo* or control (stock #145) were dissected in LAS2 buffer at 37**°**C and incubated for 10 minutes at 37**°**C. A brief 300 g centrifugation step (5 minutes) was followed by a 1 hour ultracentrifugation at 100,000 g at 37**°**C. Supernatants containing globular actin were removed and pellets resuspended in actin depolymerization buffer on ice, by pipetting up and down every 15 minutes for 1 hour. Pellets containing F-actin fractions and supernatants containing G-actin fractions were run on an SDS-PAGE gel and Western blots performed (as above) using a primary mouse monoclonal anti-actin antibody. Bands were quantified using densitometric analysis in ImageJ (as above).

## Results

### *Wolbachia* infection is lost or reduced in fly mutants heterozygous for actin binding proteins chickadee and quail

**Fig 1:**
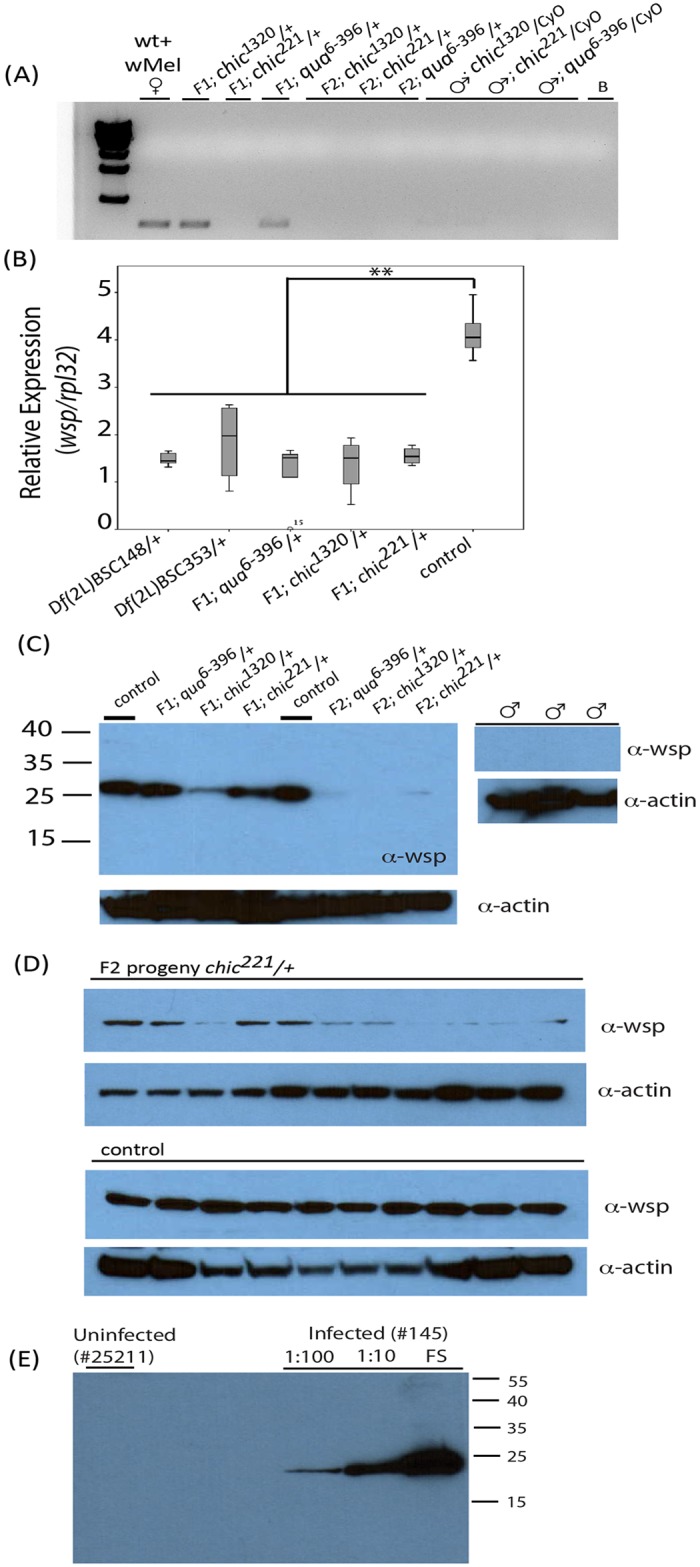
Presence of *Wolbachia* within various *Drosophila melanogaster* genotypes and their offspring assessed using polymerase chain reaction (A) and qPCR targeting the *wsp* gene on individual flies (B) or Western blot using antibodies against Wsp on both pooled fly lysates (C) and individual flies (D). Anti-actin loading controls also shown. Consistency of *Wolbachia* Wsp production in wild-type control flies shown in panel (D) using 10 age-matched female flies (stock 145). Variability in the maintenance of the *Wolbachia* infection is shown in panel (D) where F2 *chic* ^*221*^ */+* progeny are probed with anti-wsp antibody. Specificity of the anti-Wsp antibody shown in panel (E) where uninfected flies are unreactive and intensity of reactivity is directly associated with amount of fly lysate loaded (FS = full strength).

All three actin binding protein mutant fly stocks used in this study were uninfected with *Wolbachia* upon receipt from the Bloomington Drosophila Stock Center. In order to establish an infection in the flies, infected control females were crossed with mutant uninfected males to generate F1 progeny, half of which carried the mutation, and half of which carried the Cyo balancer (a second chromosome containing inversion breakpoints and a dominant visible mutation of curly wings). F1 heterozygous mutants for the actin binding protein alleles were then back-crossed to the paternal mutant line (mutant/Cyo) and F2 progeny from that cross, carrying the mutation and harboring straight wings, were collected. We screened both the F1 and F2 progeny for *Wolbachia* infection using PCR against the *Wolbachia* surface protein gene (*wsp*) (Fig 1A). We observed a trend where *Wolbachia* transmission was not complete in these crosses. For example, the bacterium could be introduced into some heterozygous mutant backgrounds; F1 progeny were infected if they resulted from crosses between control females and *chic* ^*1320*^ */CyO* as well as *qua* ^*6-396*^ */CyO* fathers, but the bacterium failed to colonize *chic* ^*221*^/+ F1 progeny efficiently. We were unable to detect *Wolbachia* in many of the F2 progeny (Fig 1A). In order to quantify this reduction in titer, we performed qPCR on DNA extracts from F1 progeny from each of five individuals from the heterozygous mutants and compared these results to the quantified *Wolbachia* loads found in control flies (Fig 1B). Progeny from each F1 cross have a statistically significant reduction in *Wolbachia* titer (as quantified through qPCR) compared to the control lines (p < 0.01 for all pairwise comparisons, using a Bonferroni correction for df = 8). As additional support for the importance of the *chic* and *qua* loci in the *Wolbachia* titer defects we observed, we also quantified the amount of *Wolbachia* within two chromosomal deficiency stocks (deletions in the same region as either *chic* or *qua* in isogenic backgrounds)[25]. These deficiencies showed the same phenotype as our *chic* and *qua* mutants, supporting our observation that these genomic loci are responsible for the *Wolbachia* titer defect (Fig 1B). In addition to reductions in the F1 progeny, we also quantified a reduction in F2 progeny for the three actin mutant lines. For flies in which we can detect *Wolbachia*, the F2 progeny are further reduced in titer compared to the F1 lines (ratio of expression F1 versus F2: min = 0.56, max = 0.78).

In order to control for effects of host genetic background on *Wolbachia* titer, we created isogenized lines from the control stock (145) and each of the mutant stocks by backcrossing to an uninfected *w; Sco/Cyo* line for three generations. We then crossed these *Wolbachia* infected F3 females (*w; Sco/Cyo)* to *Wolbachia* uninfected *w; mutant/Cyo* males (S1 Fig). In the F5 generation, we observed a significant effect of genotype on *Wolbachia* titer. Specifically, and regardless of mutant allele, mutant/Cyo progeny were reduced in *Wolbachia* titer by 1/3 compared to their *w; Sco/Cyo* siblings (mean relative ratio *wsp/rpl32*; t = -4.514; df = 9; p = 0.001). This result suggested to us that the reduction in titer was at least partially due to a result of a developmental defect in *Wolbachia* maintenance and persistence within the heterozygous mutant hosts.

As an additional control for host genetic background and to explore direct effects on profilin knockdown during development, we took advantage of an infected fly stock carrying a UAS inducible profilin-specific short hairpin silencing trigger (RNAi; stock #34523, genotype *y* ^*1*^ sc* v ^*1*^; P{TRiP.HMS00550}attP2) [27]. In order to test the effect of induction on fly development (to recapitulate the developmental lethality of the profilin null) we crossed homozygous females from this line to *w;* P{w+, Act GAL4} /TM3 males. From this cross we only recovered stubble progeny, suggesting that this particular RNAi line, which hadn’t previously been utilized in a publication to knock down profilin expression, is effective. In order to test the effect of induction on fly development we crossed homozygous females (*y* ^*1*^ sc* v ^*1*^; P{TRiP.HMS00550}attP2) to a homozygous Hsp70:Gal4 driver [2–5]. Third instar larvae were shocked at 37C for 10 minutes to induce the short hairpin and late pupae collected for RNA and DNA extraction (N = 8 for each treatment and genotype; *y* ^*1*^ sc* v ^*1*^; P{TRiP.HMS00550}attP2 with or without Hsp70:Gal4 and with or without heat shock). In the maternal *y* ^*1*^ sc* v ^*1*^; P{TRiP.HMS00550}attP2 background, heat shock did not affect either *Wolbachia* titers (t = 1.207, df = 2, p = 0.351) nor profilin expression (t = -1.144, df = 2, p = 0.371). In contrast, profilin expression was statistically significantly reduced in flies expressing the RNAi construct compared to non-heat shocked siblings (the mean expression ratio *chic/rpl32* = 0.57; t = -6.240; df = 2; p = 0.025). In addition, knockdown of profilin did have a significant and measurable effect on *Wolbachia* titers in these same flies; the fly *Wolbachia* titers were reduced by 1/3 compared to their non-heat shocked siblings (mean relative ratio *wsp/rpl32* = 0.66, t = -8.593; df = 2; p = 0.013).

To provide additional support for the reduction in titer observed via PCR, we probed Western blots of pooled or individual fly lysates produced from the F1 and F2 progeny and their parents for Wsp (Fig 1C, 1D and 1E). Results corroborated our previous finding that *Wolbachia* transmission was imperfect in the mutant flies (Fig 1A and 1B). Specifically, infected F1 progeny, especially in the *chic* mutant backgrounds, appeared to carry a reduced titer of *Wolbachia* when compared to the maternal, infected line (Fig 1). Indeed, flies from control crosses are consistently higher titer in *Wolbachia*, as based on densitometric quantitation of Western blot bands (Average +/- STERR over 5 experiments for Control = 13,106 +/- 3,294; *chic* ^*1320*^/+ = 6,418 +/- 4,890; *chic* ^*221*^/+ = 6,545 +/- 1,576; *qua* ^*6-396*^/+ = 6,179 +/- 645; t-test; p = 0.036, 0.001, 0.002 for each heterozygous mutant compared to control). Additionally, we could detect a statistically significant reduction between the F1 and F2 heterozygous mutant flies (p = 0.012). As observed in our results based on PCR, *Wolbachia* titer (based on quantity of protein on a Western blot) is also reduced, with some variability, in the F2 progeny (Fig 1D). We hypothesized that the loss of *Wolbachia* in some F2 progeny was a result of a reduction in *Wolbachia* titer in F1 females during oogenesis. We therefore visualized the *Wolbachia* infection in the germarium in F1 females (mutant/+; below).

### *Wolbachia* is reduced in the germarium and early egg chamber when hosts are heterozygous mutants in chickadee or quail

**Fig 2:**
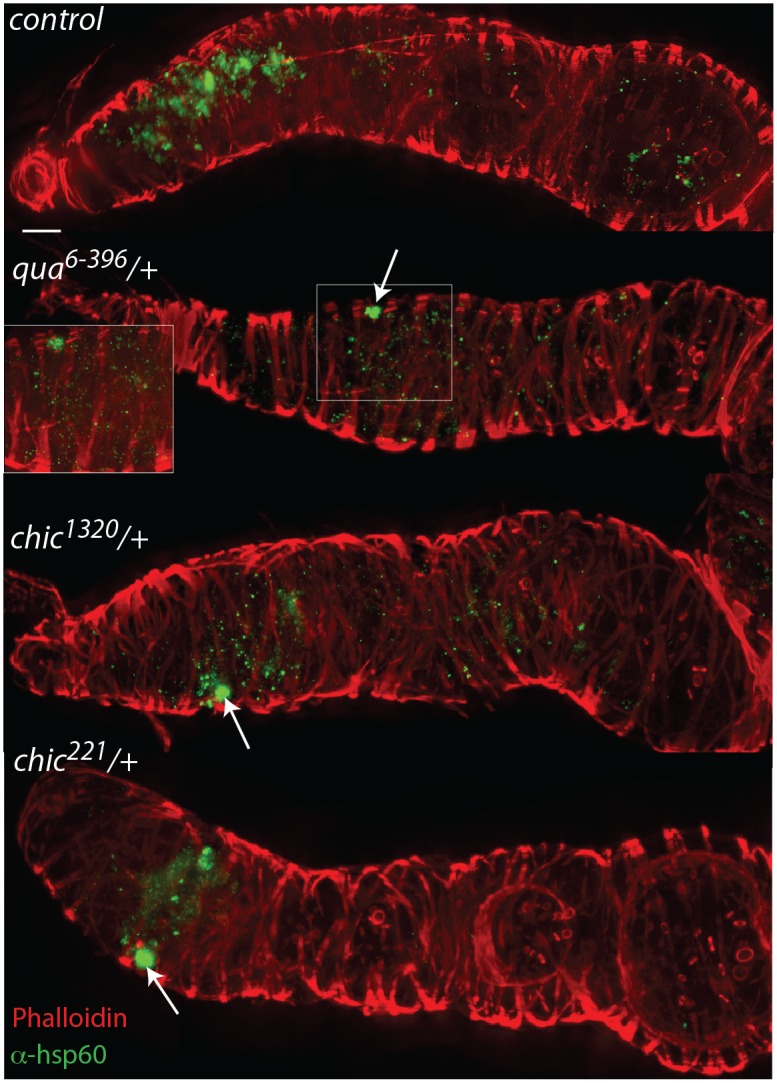
Mutations in actin binding proteins reduce the titer of *Wolbachia* within the region 2 and early egg chambers in heterozygous mutant flies. *Drosophila melanogaster* germaria from control, *qua* ^*6-396*^/+, *chic* ^*1320*^/+, and *chic* ^*221*^/+ backgrounds, stained with α-Hsp60 for *Wolbachia* (green) and Acti-stain phalloidin conjugate for actin (red). Arrows point to *Wolbachia* aggregates within heterozygous mutants. Scale bar = 10 μm. Inlays are 100x magnification of aggregates within the tissue.

**Fig 3:**
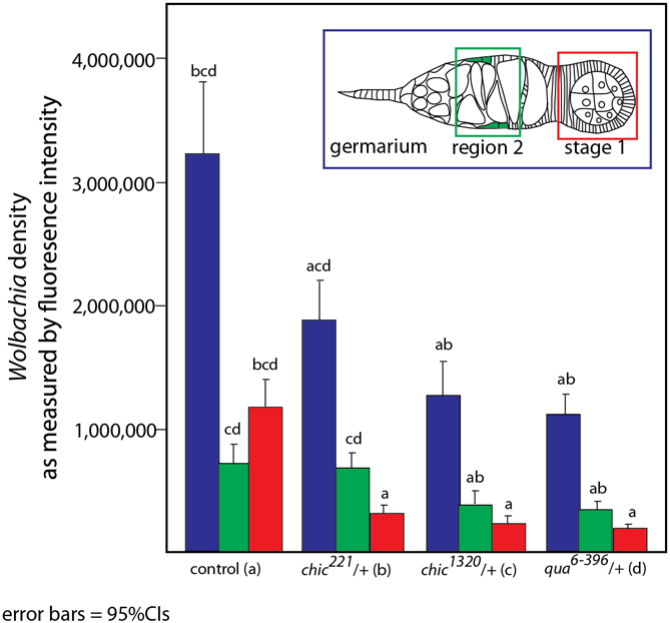
Quantification of reduction in *Wolbachia* titer during oogenesis, within the entire germarium (in blue), region 2, including the somatic stem cell niche (SSCN, green) and early egg chambers (stage 1, red). Flattened, maximum projections from z-sections were utilized to compare between control flies and *chic* ^*221*^/+, *chic* ^*1320*^/+ and *qua* ^*6-396*^/+ F1 progeny stained with Hsp-60 for *Wolbachia*. Error bars = 95% confidence intervals and statistical significance in pairwise comparisons is shown above each bar (Kruskal Wallis tests followed by pairwise Mann-Whitney U tests between genotypes; p < 0.05).

To colonize the oocyte, and therefore complete maternal transmission, *Wolbachia* occupy the germline and somatic stem cell niches (SSCN) in their hosts [18,26,31]. *Wolbachia* can achieve this localization after injection into the fly abdomen, suggesting that the stem cell niche targets are essential for *Wolbachia* infection [31]. The *Drosophila* ovariole provides an opportunity to view oocyte development and *Wolbachia* localization within each progressive stage. *Wolbachia* concentrate preferentially in the somatic stem cell niche, which is thought to serve as a source of infection for the germline. As germline development progresses from regions 2a to 2b, *Wolbachia* are thought to infect via the somatic stem cell niche, increasing the numbers of bacteria found within the germline *after* association with the SSCN [31]. We utilized immunohistochemistry to detect *Wolbachia* in the germarium of our flies, producing localizations expected based on previous publications [15,18,26,31](S1 Table and S2 and S3 Figs). *Wolbachia* infection within the entire germarium is significantly reduced in heterozygous mutant flies (when comparing the amount of fluorescence observed in control flies to that found in either *chic* ^*221*^ */+*, *chic* ^*1320*^ */+* or *qua* ^*6-396*^ */+*, respectively; Mann-Whitney U = 171.5, Z = -3.995, p < 0.001; Mann-Whitney U = 98, Z = -5.295, p < 0.001; Mann-Whitney U = 55.5, Z = -5.496, p < 0.001; Figs 2 and 3). When either *chic* ^*1320*^ */+* or *qua* ^*6-396*^ */+* heterozygous mutant flies are infected, the *Wolbachia* titer in region 2 (as quantified by anti-Hsp60 staining) is also significantly reduced, compared to the control maternal line (Mann-Whitney U = 194, Z = -3.78, p < 0.001; Mann-Whitney U = 134; Z = -4.097, p < 0.001, pairwise comparison between control and *chic* ^*1320*^ */+* or *qua* ^*6-396*^ */+* heterozygous mutants, respectively; Fig 3). Additionally, *Wolbachia* infection within early egg chambers (stage 1) is significantly reduced in all heterozygous mutant flies (when comparing the amount of fluorescence observed in control flies to that found in either *chic* ^*221*^ */+*, *chic* ^*1320*^ */+* or *qua* ^*6-396*^ */+*, respectively; Mann-Whitney U = 74, Z = -5.74, p < 0.001; Mann-Whitney U = 58, Z = -5.872, p < 0.001; Mann-Whitney U = 39, Z = -5.767, p < 0.001; Figs 2 and 3). In order to quantify this reduction, for each germarium, we calculated the ratio of fluorescence intensity in the earliest egg chamber over that found in region 2 (as quantified by anti-Hsp60 staining). Each of the three mutant lines showed a statistically significant reduction in this ratio when compared to control germaria (average ratios for control flies: 1.72; *chic* ^*221*^ */+*: 0.52*; chic* ^*1320*^ */+*: 0.64; *qua* ^*6-396*^ */+*: 0.80, t-test; p < 0.0001). The reductions in infection in the germaria suggest two things: (1) that *Wolbachia* has difficulties in transiting or maintenance in a population within the germarium during development in the heterozygous mutant flies and (2) even when region two, the location of the SSCN, is occupied by *Wolbachia*, the bacteria are deficient in colonization of the early egg chamber in the heterozygous mutant flies (Fig 3). We did not quantify differences in staining of the presumed germline stem cell niche due to variability in staining in this region within the control flies.

Within heterozygous mutant flies, we observed that the *Wolbachia* that successfully manage to colonize the germarium do so with a distinctive localization; these *Wolbachia* appear as aggregates, in sharp contrast to the more even distribution of *Wolbachia* within control germaria (Table 1 and Fig 2). Under high magnification (100x), the *Wolbachia* aggregates within the heterozygous mutant flies appear to be multiple *Wolbachia* forming micro-colonies within the tissue, based on shape and size and consistent localization within the genotypes.

**Table 1:** *Wolbachia* aggregates within the germarium of *Drosophila melanogaster*.

	Aggregate observed	Wild-type distribution	N	% Aberrant
Control	2	28	30	6.7%
*chic* ^*1320*^/+	17	21	38	44.7%
*chic* ^*221*^/+	16	19	35	45.7%
*qua* ^*6-396*^/+	25	15	40	62.5%

Distinctive aggregates observed predominantly in heterozygous mutant flies (N ≥30).

### Characterization of F/G-actin and profilin expression within heterozygous mutant flies

Both *Drosophila* profilin (*chickadee*) and villin (*quail*) are important in the regulation of F-actin during oogenesis. Because profilin promotes the polymerization of F-actin filaments and villin stabilizes these filaments through bundling, we were curious to know whether or not the heterozygous mutant flies differed in the quantity of F-actin found in the germarium, when compared to control flies. In addition, visualization of the F-actin cytoskeleton allowed us to examine the actin ring canals in the heterozygous mutant flies at all stages of oocyte development. At no point were ring canals occluded by nuclei, supporting our finding that cytoplasmic streaming and maternal dumping are unaffected in heterozygous mutant flies (N = 300, scored by eye). Using quantified fluorescence of F-actin in the images we were unable to detect a statistically significant difference between median levels of phalloidin staining in control flies compared to the heterozygous mutants (Kruskal Wallis test: χ2 = 4.005, df = 3, p = 0.261; S4A Fig). Because F-actin levels in the germarium (observed through phalloidin staining) did not correlate with *Wolbachia* intensity (as quantified by anti-Hsp60 staining), the *Wolbachia* titer phenotype observed in these flies may not be directly related to the F-actin network in the germarium. We therefore examined the *in vivo* amounts of filamentous and globular actin in ovaries from control flies and compared this to that seen in heterozygous mutant *chic* ^*221*^ flies. Using ultracentrifugation coupled to Western blot, we found that we could consistently detect globular actin in the ovaries of control flies. In contrast, we found a statistically significant decrease in total amount of globular actin detected in the heterozygous mutant lines (Kruskal Wallis: χ^2^ = 4.192, df = 1, p = 0.041; S4B Fig).

The difference in G actin between control and heterozygous *chic* ^*221*^ flies prompted us to investigate expression of profilin in the *chic* ^*221*^ */*+ F1 mutants and control flies. The rationale was that although these flies are phenotypically wild type, the dosage effect of a single, wild type chromosome in the *chic* ^*221*^ */+* F1 mutants might be significant and correlate with *Wolbachia* absence. We extracted both RNA and DNA from individual F1 *chic* ^*221*^ */*+ female flies as well as age-matched control flies and used quantitative RT-PCR to detect profilin transcript levels (in total RNA) and *Wolbachia* surface protein (in total DNA) relative to Rpl32. Control flies express, on average, 2x as much profilin as heterozygous mutant F1 progeny (means control μ = 4.03; mutant μ = 2.28; t = 2.590, df = 11.31, p = 0.025). Additionally, although we could detect *Wolbachia* in each of the wild type flies included (N = 10), we were only able to detect a *Wolbachia* infection in three of the heterozygous mutant F1 flies (S4C Fig). *Wolbachia* may have been present in these flies, but at titers below the limit of detection for this method.

### Heterozygous mutants in chickadee and quail produce the same number of progeny as control flies and lay eggs of normal size and shape

Because *Wolbachia* target the germline, and within the germarium, the stem cell niche [18,31], the number of egg chambers produced by the host may affect *Wolbachia’s* ability to be transmitted between generations. Flies that are *homozygous* mutants in *chickadee* show defects in germline stem cell proliferation as well as enclosure by somatic cyst cells [32,33] so it was therefore important to confirm that the *heterozygous* mutant flies do not display similar defects. We counted the number of viable progeny resulting from individual crosses within mutant fly lines and compared the number of resulting offspring to those from control crosses. Heterozygous villin or profilin mutant flies do not show a defect in fertility when compared to control flies (S5 Fig). Additionally, we observed over 300 eggs for each of the fly mutant stocks and did not see any morphological abnormalities when compared to the control stock (N = 300, scored by eye).

## Discussion

*Wolbachia* maternal transmission in *Drosophila melanogaster* is normally extremely effective, with perfect transmission observed in laboratory populations and near perfect transmission in the wild [10–12]. *Wolbachia* are thought to localize in the germarium, and ultimately in the oocyte, in order to accomplish this maternal transmission. Previous work has shown that *Wolbachia* use host microtubules to localize preferentially to the oocyte during development [15–17]. The striking anterior localization of *Wolbachia* during oogenesis can be perturbed by feeding *Drosophila* microtubule inhibitors such as colchicine, or by mutations that perturb the microtubule cytoskeleton [15]. In contrast, direct treatment of dissected ovaries with actin disrupting drugs (such as cytochalasin-D) does not alter this localization [15]. However, other pieces of evidence suggest that *Wolbachia* manipulate the host actin cytoskeleton. For example, *Wolbachia* injected into the abdominal cavity of *Drosophila* migrate to the germline stem cell niche, a feat that requires traversing several host tissues and cell types [31]. Also, *Wolbachia* in the terrestrial isopod *Armadillidium vulgare* are not found in all primary oocytes and instead, enrichment of *Wolbachia* is seen during the course of development [34]. Finally, *Wolbachia* are associated with areas of weak cortical actin staining in filarial nematodes, suggestive of a mechanism for entry into the germline from somatic cells [17,19]. Therefore, it is likely that *Wolbachia* use both microtubules and actin for persistence in the host and maintenance across host generations.

Oogenesis in *Drosophila* relies on rearrangements of both the actin and microtubule networks [35]. We were therefore careful in our analysis to separate direct effects of actin modulation from indirect effects resulting from perturbations of the reproductive biology of the fly. Products of both the quail and chickadee loci are necessary for fly reproduction [22,36–38]; homozygous or hemizygous mutants in either gene result in fertility defects or are lethal. Importantly, in this study we followed *Wolbachia* infections in phenotypically wild type flies harboring a functional copy of the actin binding protein in question. These heterozygous mutant flies produce the same number of offspring as the control flies and produce eggs with the same morphology as controls, however the flies do not faithfully maintain a *Wolbachia* infection. Several hypotheses partially explain our data, and below we delineate our hypothesis and alternative hypotheses and summarize our evidence to support or refute them.

### Heterozygous mutant flies are phenotypically wild type with respect to oocyte polarization and number of progeny

The developing oocyte is loaded with maternal determinants (e.g. mRNA and protein), a process which begins early (stage 1), and continues until about stage 10 when maternal nurse cells dump their remaining cytoplasmic contents into the oocyte [35]. The actin cytoskeleton is critical to this process, as mutations in actin binding proteins have been known to cause severe defects. Specifically, cytoplasmic actin bundles are required to restrain the nurse cell nuclei during transport; mutations in *quail*, which regulates bundling of cytoplasmic actin, cause a dumpless phenotype [39,40]. In *quail* mutant flies, nurse cell nuclei can be observed extending through the actin ring canals [39]. We reasoned that although heterozygous mutant flies (*chic* ^*221*^/+, *chic* ^*1320*^/+ and *qua* ^*6-396*^/+) produce viable progeny, and we found no occluded ring canals in any of these backgrounds, a subtle defect in maternal cytoplasmic dumping could alter the ability of *Wolbachia* to be transmitted faithfully to the oocyte. *Wolbachia* has been suggested to utilize cytoplasmic dumping to increase titer in the oocyte (as compared to the nurse cells) [15]. In addition to regulating the bundling of microtubules and therefore cytoplasmic streaming, profilin is also required for posterior patterning in the oocyte as *chic* mutants fail to localize STAUFEN and *oskar* mRNA [41]. *Wolbachia* utilizes these posterior determinants to localize in the oocyte, as disruption of *osk* and *stau* results in mislocalization of *Wolbachia* in *D*. *melanogaster* [16]. If heterozygous mutant flies are defective in cytoplasmic dumping or polarization, we should observe both egg size and morphology defects. Over 300 eggs were scored for each of the mutant lines, as well as control flies, without any phenotypic differences detected. Importantly, however, the primary loss of *Wolbachia* in these heterozygous mutants occurs in the germarium, before defects would begin to affect *Wolbachia* titers. Therefore, although our fly mutants could conceivably exhibit subtle polarization defects, these defects alone would not entirely explain the observed phenotype.

In addition to serving important roles during maternal loading in the late stage oocyte, profilin functions in germline stem cell (GSC) maintenance and germ cell enclosure by somatic cyst cells [32,33]—homozygous *chickadee* mutants fail to maintain germline stem cell number. However, *chic* ^*221*^/+ flies are equivalent to wild type [32]; that is to say, heterozygous mutant flies do not have a GSC deficiency. Importantly, although *Wolbachia* are known to alter germline stem cell proliferation [26] and some *Wolbachia* colonize the germline stem cell niche [18], *w*Mel colonizes the somatic stem cell niche in *Drosophila melanogaster* (Fig 2). Regardless, a defect in fertility, resulting from defects in GSC maintenance might affect *Wolbachia* proliferation in these mutant flies. We therefore counted the number of viable progeny (a measure of fertility) for each of the mutant lines. No statistically significant difference was observed for any of the heterozygous, mutant flies, when compared to the control (S5 Fig). We therefore did not find support for this hypothesis to explain the *Wolbachia* clearing phenotype of profilin and villin heterozygous mutants.

### *Wolbachia* localization during development impacts maternal transmission

There is significant evidence that *Wolbachia* colonize the primordial germ cells and the posterior pole of developing embryos in numerous insect hosts. In *D*. *melanogaster*, for example, strain *w*Mel concentrates at the posterior pole in a poleplasm dependent fashion [16,42,43]. However, this posterior concentration of *Wolbachia* is not universal in insects nor in *Drosophila*. *Wolbachia* strain *w*Ri infects the entire embryo uniformly while B group *Wolbachia* actually show exhibit anterior localization [14]. Similarly, in other *Drosophila* species there are different patterns of *Wolbachia* colonization: although *w*Wil infects primordial germ cells, *w*Au infects the entire embryo [44]. This posterior localization is clearly important—the extent of CI is correlated with the number of *Wolbachia* in the posterior of the embryo [14]. However, this posterior localization is not necessarily correlated with maternal transmission, which is near 100% for some *Drosophila* species and quite low for others [45–47]. This result suggests that high titer localization to primordial germ cells and the posterior pole does not guarantee maternal transmission. However, if our heterozygous mutant flies induce defects in these early localization patterns (to the posterior pole or to the developing germ line), we might expect the inefficient transmission phenotype observed.

What other ways might *Wolbachia* use to eventually colonize the germline? *Wolbachia* colonization of somatic tissues has been known for some time [48] but recently, it has been suggested that *Wolbachia* infection of the soma may serve as a reservoir for germline infection. In the terrestrial isopod, *Armadillidium vulgare*, *Wolbachia* is absent from many early oocytes and infects the older oocytes late in development, an enrichment that is thought to come from a somatic reservoir (the follicle cells) [34]. In nematodes, *Wolbachia* initially are concentrated in the posterior of the P_2_ blastomere, the precursor of the adult germ line. However, *Wolbachia* are subsequently excluded from the germ line in the next cell division and instead, invade the germ cells later, from the surrounding somatic gonadal cells [19]. This soma to germ cell invasion in *Brugia* is correlated with a disruption in polymerized actin at those foci [19]. Because we observed a reduction in anti-Hsp60 staining in stage 1 egg chambers of heterozygous mutant flies as well as transmission defects, one interpretation of our data is that *Wolbachia* require actin for soma to germline transmission. Importantly, however, we did not observe actin disruptions (similar to those seen in *Brugia*) within *Drosophila* germaria.

### Actin regulation impacts *Wolbachia* titers during development, affecting transmission efficiency

Our data suggest that *Wolbachia* rely on the actin cytoskeleton to achieve adequate titer in the *Drosophila* host during development. First, we observe reductions in titer of *Wolbachia* in heterozygous mutants compared to both their non-mutant sibling controls as well as parental controls (Fig 1). Second, knockdown of profilin in third instar larvae reduces *Wolbachia* titer in pupae, suggesting that the regulation of actin is important to the maintenance of a *Wolbachia* infection during development. Additionally, passage of *Wolbachia* through heterozygous mutant lines for multiple generations results in the enrichment for mutant *Wolbachia;* the heterozygous mutant flies bottleneck the *Wolbachia* infection, increasing the stochastic segregation of variants [49]. This decrease in titer may explain the inefficient transmission of *Wolbachia* observed in the mutant flies. Actin may be used by *Wolbachia* to properly localize during development, or may support the infection via other unknown mechanisms.

### Potential mechanisms to explain the *Wolbachia* phenotype in mutant flies

Both of the proteins investigated here (profilin and villin), are known to increase the amount and stability of F-actin in the *Drosophila* egg chamber. Profilin promotes F- actin in the follicular epithelium while villin bundles and binds to filamentous actin [37,50]. One potential cause of the *Wolbachia* phenotype in these backgrounds is a mis-regulation in F-actin content. Interestingly, *chic* mutants have been previously observed to exhibit decreased F-actin levels in the follicle cells [50]. Both the somatic stem cell niche and the follicular epithelium have been suggested to be a source of *Wolbachia* during oogenesis [18,34]. Because *Wolbachia* densely colonize the follicular epithelium tissue, and because it surrounds the oocyte throughout development, this tissue may be a candidate for the source of the infection. We detected a significant reduction in the amount of actin in heterozygous mutant *chic* ^*221*^ flies compared to controls, which corresponded to a decrease in profilin transcripts and a decrease in detected *Wolbachia* (S4 Fig). These data are suggestive of a role for actin in *Wolbachia* maintenance and transmission but do not elucidate an exact mechanism.

We have shown that the host actin cytoskeleton is clearly important for the maintenance of a *Wolbachia* infection. Perhaps this reproductive parasite secretes proteins that interact directly with eukaryotic actin or host actin binding proteins. Indeed, other members of the *Rickettsiales* are known for their striking coopting of host actin in the production of comet tails [51]. However, when intracellular, *Wolbachia* persist within membrane-bound compartments and no such comet-like structures have been observed to be associated with the vacuole [21]. That said, our results here and the work of others strongly suggest that *Wolbachia* is able to enter and exit eukaryotic cells; *Wolbachia* transit to the germline from the fly abdomen and are loaded into the germ cells from surrounding somatic cells [18,26,31]. *Wolbachia’s* success likely depends upon an ability to secrete proteins that modify host actin to promote internalization by non-phagocytic cells. Recently, *in vitro* biochemical associations between the filarial nematode *Wolbachia* (*w*Bm) PAL-like protein *wBm0152* and actin have been observed, although results do not conclusively implicate this particular protein in interactions with host actin during infection [20]. Regardless, as is clear from our work, a *Wolbachia* infection depends on the actin cytoskeleton. Therefore, future work to identify and characterize *Wolbachia* proteins that bind to or alter host actin dynamics will be important for understanding the molecular basis of the interaction between the host and the symbiont.

### Summary

In order for intracellular, maternally transmitted symbionts to successfully infect the next generation, the bacteria must target the oocyte. *Wolbachia* achieves this through a specific infection of the somatic stem cell niche in the germarium of *Drosophila melanogaster* [18]. Here we show that *Wolbachia* is extraordinarily sensitive to the regulation of actin, such that phenotypically wild type heterozygous mutant flies cannot faithfully transmit the bacterium to their progeny. Our results, particularly that titer is significantly reduced in the germaria of *chic* ^*221*^/+, *chic* ^*1320*^/+, and *qua* ^*6-396*^/+ flies, suggest that *Wolbachia* utilize host actin to enter and persist within host tissues during *Drosophila* development. Additionally, our finding that these heterozygous mutant flies cannot transmit the infection suggests that *Wolbachia* titers within a host are reduced when actin regulation is disrupted, impacting transmission efficiency.

## Supporting Information

S1 Table Total number of flies and germaria fixed, stained, and imaged for anti-Hsp60 intensity quantification as proxy for *Wolbachia* titer. (DOCX)

S1 Fig (A) Crosses used to isogenize genetic backgrounds utilized in this study and (B) experimental crosses used to compare between heterozygous mutants and w/w; Sco/Cyo sibling controls. (TIF)

S2 Fig Visual masks showing demarcated regions used in the analysis of anti-Hsp60 intensity as proxy for *Wolbachia* titer in the germarium. Blue = entire germarium; Green = region 2; Red = Stage 1 egg chamber.
(TIF)

S3 Fig Anti-Hsp60 staining of control (stock #145) germaria highlights density of *Wolbachia* in region two of the germarium and the early egg chamber. This staining is recapitulated by fluorescence *in situ* hybridization (using the Eub338-Alexa488 probe) as well as staining with another anti-body (custom anti-FtsZ). Note strong staining in the germarium and in the presumed early oocyte.
(TIF)

S4 Fig (A) Quantification of amount of F-actin in *Drosophila melanogaster* germaria during oogenesis, within the entire germarium. Maximum projections, generated from z-stacks, were utilized to compare between control flies and *qua* ^*6-396*^/+, *chic* ^*1320*^/+, and *chic* ^*221*^/+ F1 *Wolbachia* infected progeny with regards to amount of F-actin staining (using Acti-stain 488 phalloidin). Bars = minimum and maximum values. Box = first and third quartiles while the median is shown as a band through the box. Although the 95% confidence intervals overlap for all genetic backgrounds, the distributions of values for the mutants are much more variable than found in the control flies (Standard deviations = control = 1.7e6, *qua* ^*6-396*^/+ = 2.4e6, *chic* ^*1320*^/+ = 2.2e6, and *chic* ^*221*^/+ = 2.1.e6). (B) Quantification of G-actin in the ovaries of control and *chic* ^*221*^ */Cyo* female flies. Densitometry measures using western blots (a-actin) showed statistically significant reductions in actin in heterozygous mutant female flies (χ2 = 4.192; df = 1; p = 0.041) (C) Relative quantification of profilin transcripts within individual *chic* ^*221*^/*+* F1 female flies as well as wild type, control flies (stock #145). A statistically significant decrease in profliin expression was observed in the heterozygous mutant flies compared to controls (means control μ = 4.03; mutant μ = 2.28; t = 2.590; df = 11.31; p = 0.025). Importantly, *Wolbachia* was only detected in three of the twenty heterozygous mutant flies but consistently found in all of the wild type flies (using qPCR on *wsp*).
(TIF)

S5 Fig *Drosophila melanogaster* flies do not show defects in fertility when heterozygous mutant for villin or profilin. Number of viable progeny produced by control and mutant lines (villin mutant *qua* ^*6-396*^and profilin mutants *chic* ^*221*^ and *chic* ^*1320*^). In each case, 50 single pair crosses between virgin females and males from the same background were performed, parents were transferred to new vials and the offspring counted every four days. Median, quartiles and minimum and maximum number of progeny shown for each. The 95% confidence intervals overlap for all genetic backgrounds and comparisons of means are not statistically significant (95% CIs for control: 36.24–58.02; *qua* ^*6-396*^: 38.12–55.26; *chic* ^*221*^: 54.81–74.66; *chic* ^*1320*^: 23.07–44.49).
(TIF)
