# Supplementary material for: Wolbachia Utilize Host Actin for Efficient Maternal Transmission in Drosophila melanogaster
Source: PLoS Pathog. 2015 Apr 23;11(4):e1004798. doi: 10.1371/journal.ppat.1004798 (PMC4408098; doi:10.1371/journal.ppat.1004798)
Supplement: S1 Table — (DOCX) [file ppat.1004798.s001.docx]

**Table S1**. Total number of flies and germaria fixed, stained, and imaged for anti-Hsp60 intensity quantification as proxy for *Wolbachia* titer.

| Genotype | Number of flies | Number of Germaria imaged |
| --- | --- | --- |
| Control | 25 | 30 |
| *chic^221^/+* | 28 | 35 |
| *chic^1320^/+* | 20 | 38 |
| *qua^6-396^/+* | 25 | 40 |
