# Supplementary figures and images for: Wolbachia Utilize Host Actin for Efficient Maternal Transmission in Drosophila melanogaster
Source: PLoS Pathog. 2015 Apr 23;11(4):e1004798. doi: 10.1371/journal.ppat.1004798 (PMC4408098; doi:10.1371/journal.ppat.1004798)

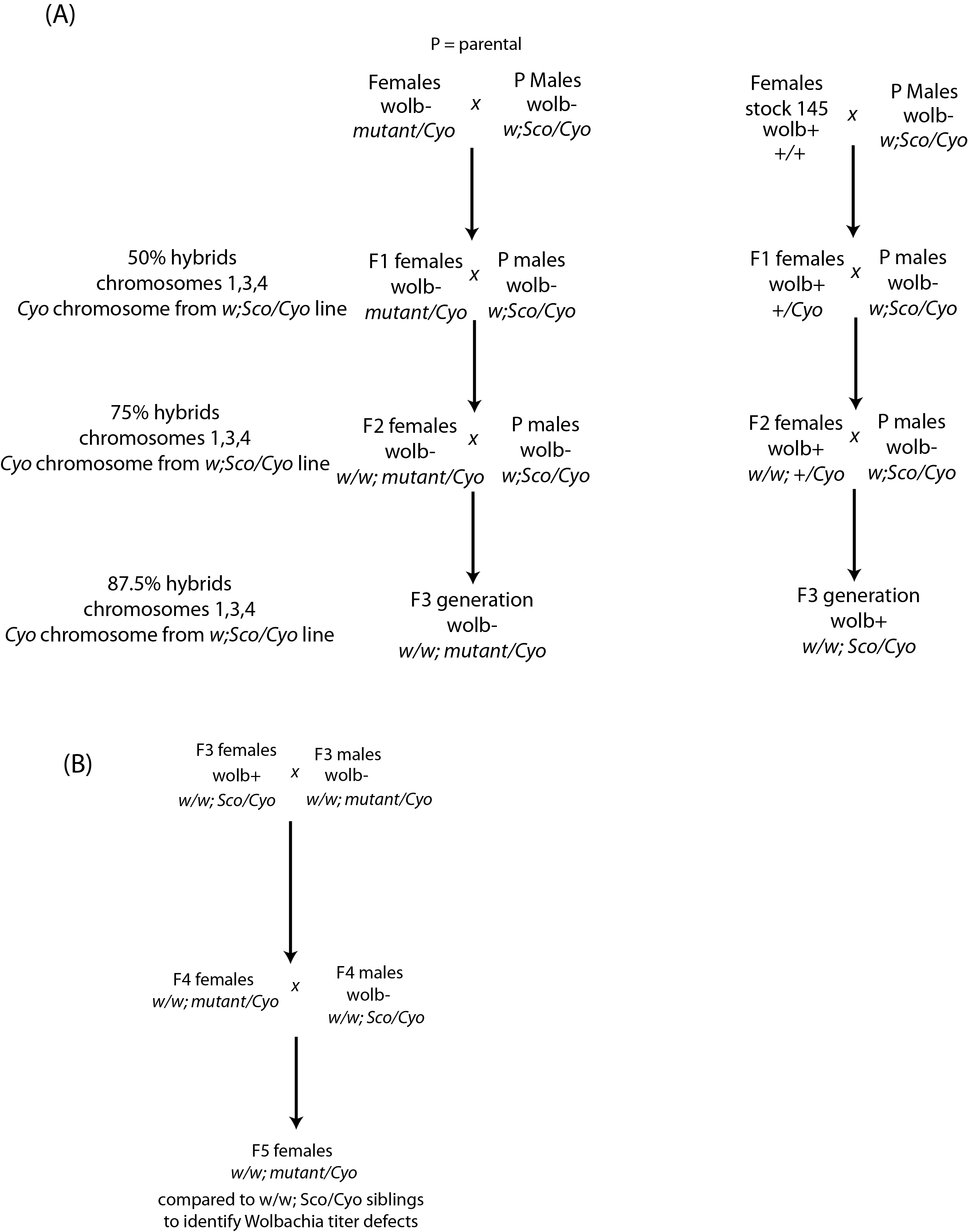

Supplement: S1 Fig — (TIF) [file ppat.1004798.s002.tif]

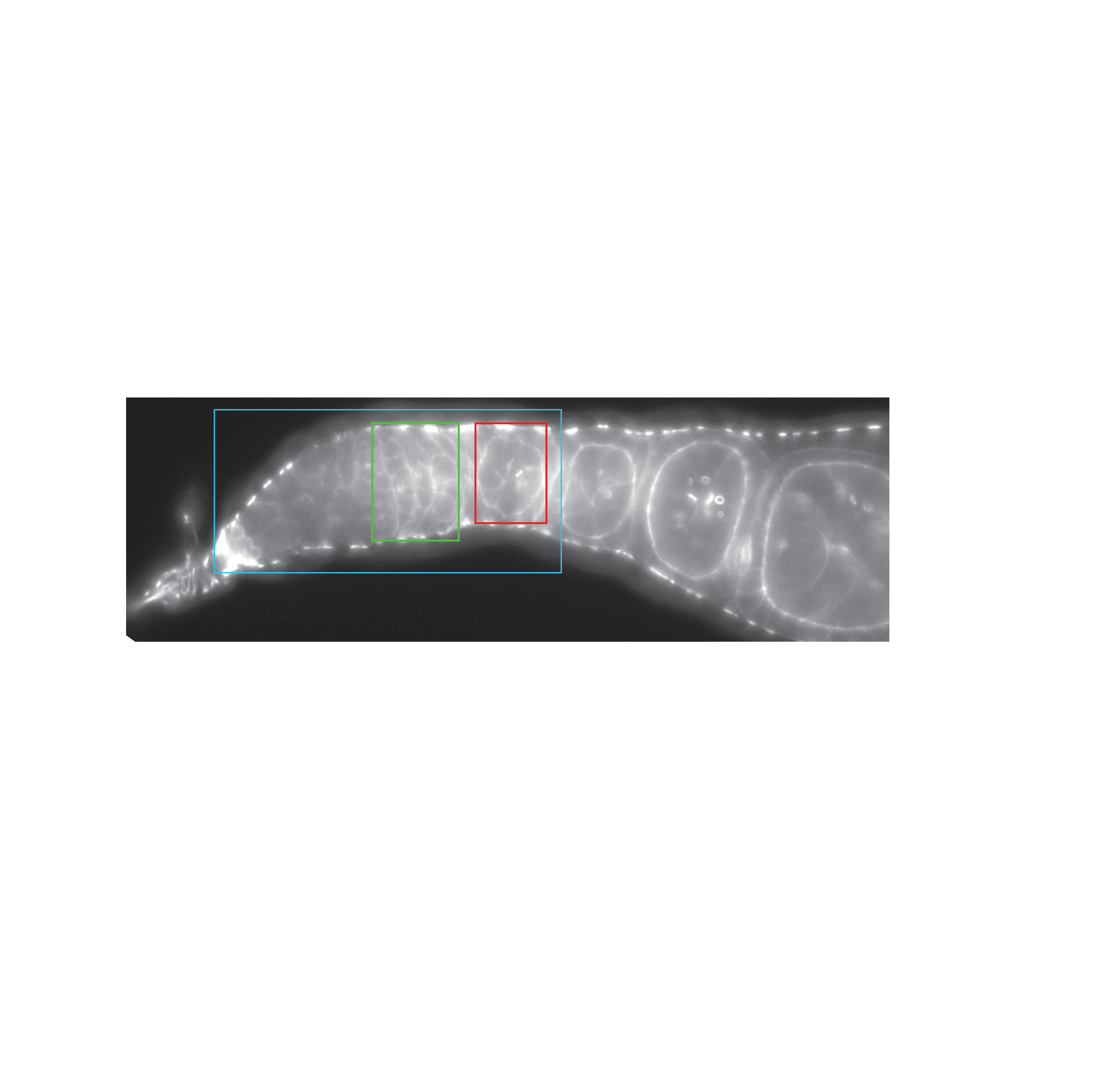

Supplement: S2 Fig — Blue = entire germarium; Green = region 2; Red = Stage 1 egg chamber. (TIF) [file ppat.1004798.s003.tif]

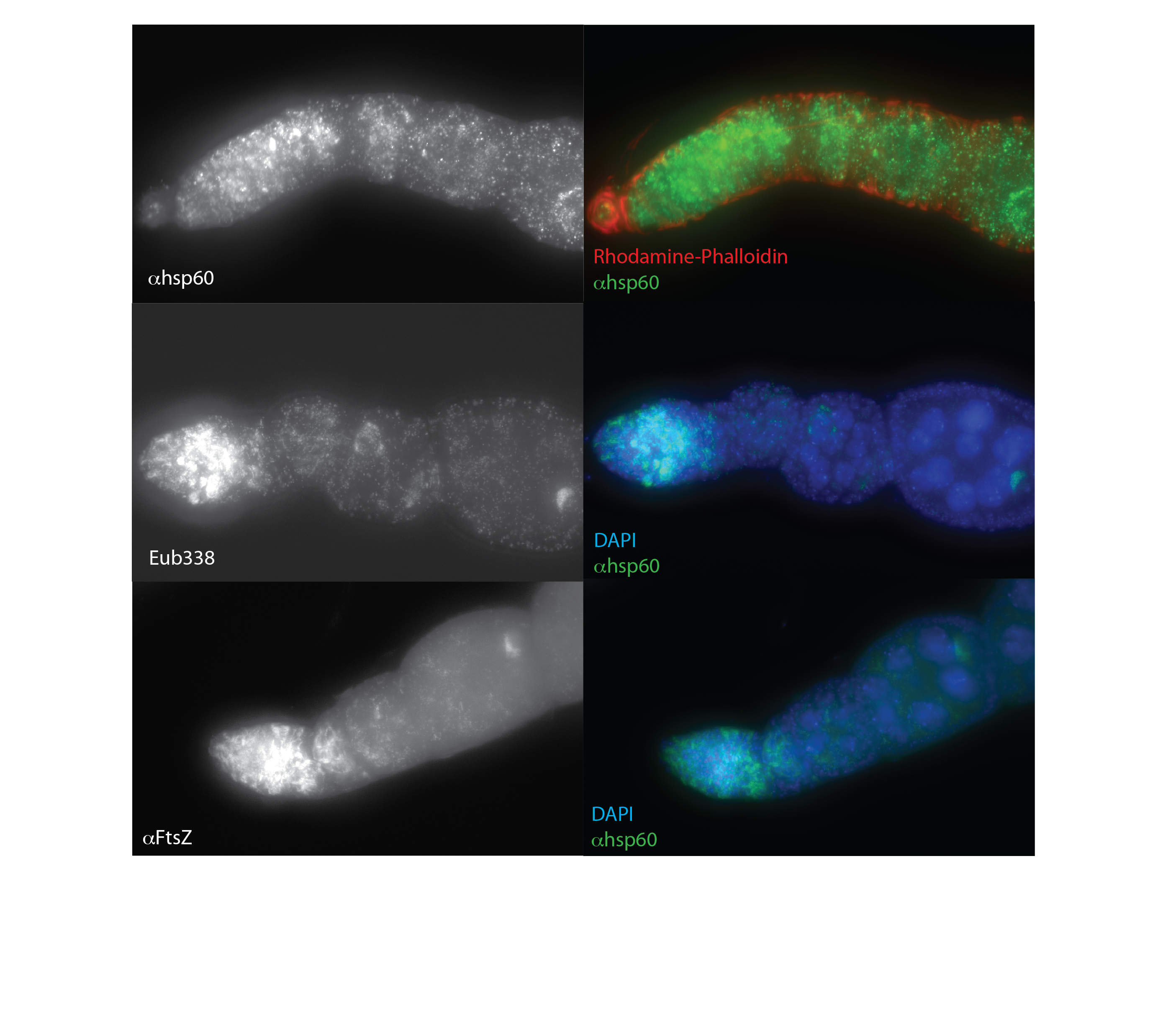

Supplement: S3 Fig — This staining is recapitulated by fluorescence in situ hybridization (using the Eub338-Alexa488 probe) as well as staining with another anti-body (custom anti-FtsZ). Note strong staining in the germarium and in the presumed early oocyte. (TIF) [file ppat.1004798.s004.tif]

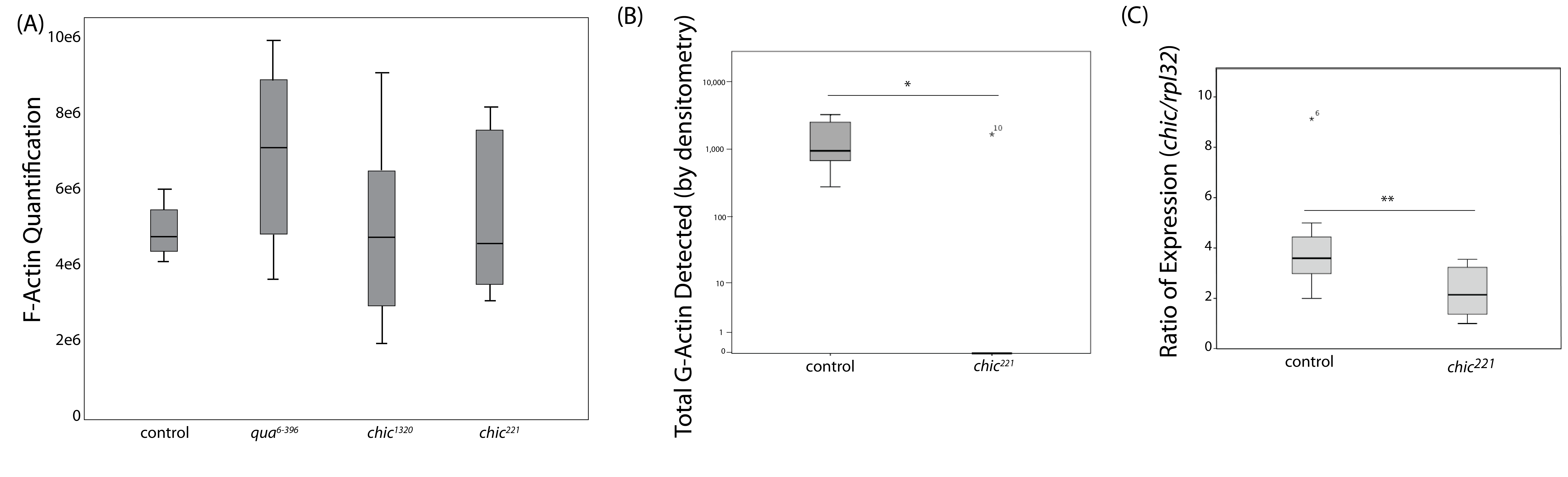

Supplement: S4 Fig — Maximum projections, generated from z-stacks, were utilized to compare between control flies and qua 6-396/+, chic 1320/+, and chic 221/+ F1 Wolbachia infected progeny with regards to amount of F-actin staining (using Acti-stain 488 phalloidin). Bars = minimum and maximum values. Box = first and third quartiles while the median is shown as a band through the box. Although the 95% confidence intervals overlap for all genetic backgrounds, the distributions of values for the mutants are much more variable than found in the control flies (Standard deviations = control = 1.7e6, qua 6-396/+ = 2.4e6, chic 1320/+ = 2.2e6, and chic 221/+ = 2.1.e6). (B) Quantification of G-actin in the ovaries of control and chic 221 /Cyo female flies. Densitometry measures using western blots (a-actin) showed statistically significant reductions in actin in heterozygous mutant female flies (χ2 = 4.192; df = 1; p = 0.041) (C) Relative quantification of profilin transcripts within individual chic 221/+ F1 female flies as well as wild type, control flies (stock #145). A statistically significant decrease in profliin expression was observed in the heterozygous mutant flies compared to controls (means control μ = 4.03; mutant μ = 2.28; t = 2.590; df = 11.31; p = 0.025). Importantly, Wolbachia was only detected in three of the twenty heterozygous mutant flies but consistently found in all of the wild type flies (using qPCR on wsp). (TIF) [file ppat.1004798.s005.tif]

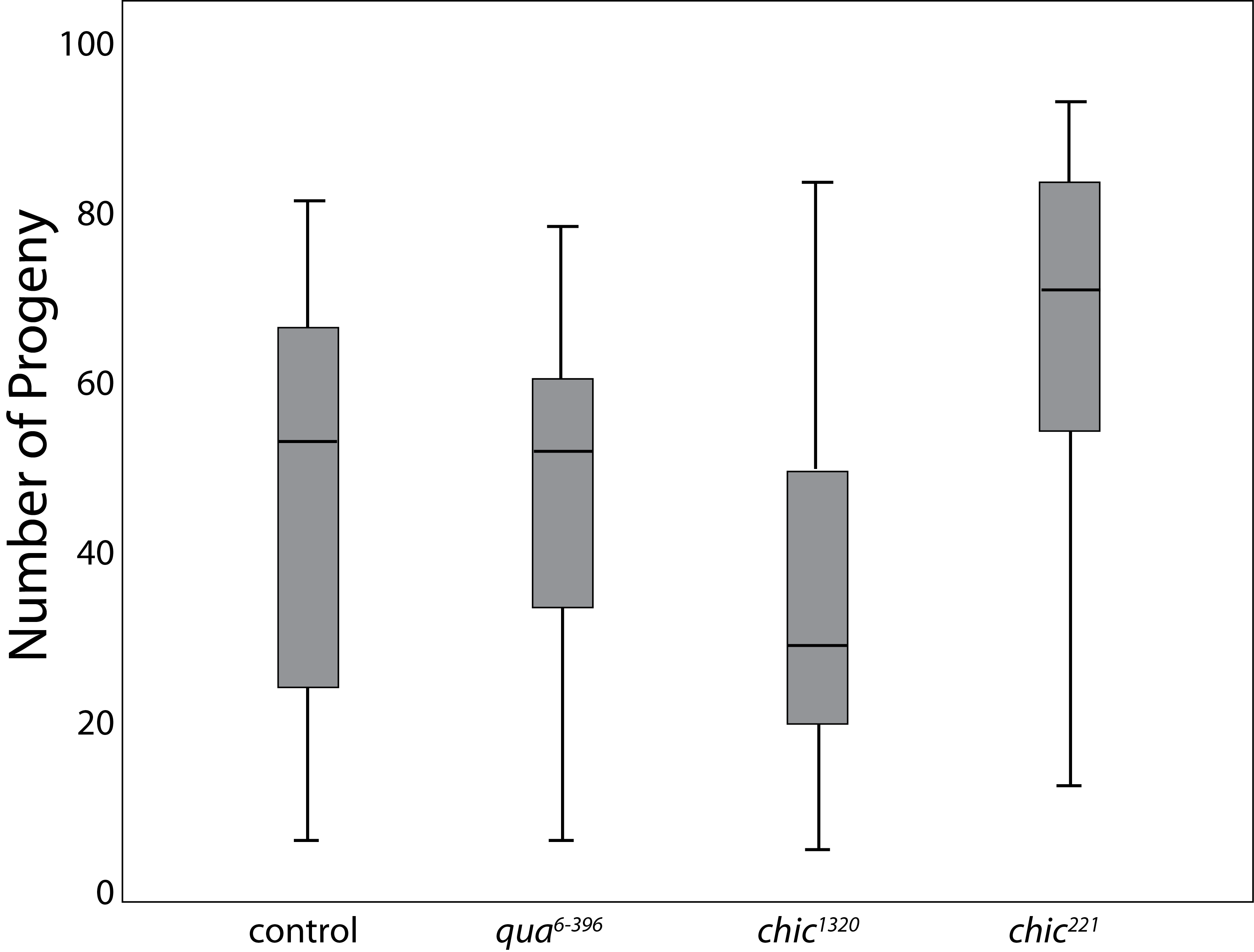

Supplement: S5 Fig — Number of viable progeny produced by control and mutant lines (villin mutant qua 6-396and profilin mutants chic 221 and chic 1320). In each case, 50 single pair crosses between virgin females and males from the same background were performed, parents were transferred to new vials and the offspring counted every four days. Median, quartiles and minimum and maximum number of progeny shown for each. The 95% confidence intervals overlap for all genetic backgrounds and comparisons of means are not statistically significant (95% CIs for control: 36.24–58.02; qua 6-396: 38.12–55.26; chic 221: 54.81–74.66; chic 1320: 23.07–44.49). (TIF) [file ppat.1004798.s006.tif]
